# High‐resolution imaging sheds new light on a multi‐tier symbiotic partnership between a “walking” solitary coral, a sipunculan, and a bivalve from East Africa

**DOI:** 10.1002/ece3.8633

**Published:** 2022-03-08

**Authors:** Natalia Herrán, Gita R. Narayan, Steve S. Doo, André Klicpera, André Freiwald, Hildegard Westphal

**Affiliations:** ^1^ Leibniz Centre for Tropical Marine Ecology (ZMT) Bremen Germany; ^2^ Department of Geosciences (FB5) University of Bremen Bremen Germany; ^3^ 28389 Leibniz Institute for Baltic Sea Research Warnemünde Warnemünde Germany; ^4^ King Abdullah University of Science and Technology (KAUST) Thuwal Saudi Arabia; ^5^ Microtrac Retsch GmbH Bitterfeld Germany; ^6^ Senckenberg am Meer (SaM) Wilhelmshaven Germany

**Keywords:** climate change, coral reefs, solitary coral, symbiosis, turbid reefs, Western Indian Ocean

## Abstract

Marine symbioses are integral to the persistence of ecosystem functioning in coral reefs. Solitary corals of the species *Heteropsammia cochlea* and *Heterocyathus aequicostatus* have been observed to live in symbiosis with the sipunculan worm *Aspidosiphon muelleri muelleri*, which inhabits a cavity within the coral, in Zanzibar (Tanzania). The symbiosis of these photosymbiotic corals enables the coral holobiont to move, in fine to coarse unconsolidated substrata, a process termed as “walking.” This allows the coral to escape sediment cover in turbid conditions which is crucial for these light‐dependent species. An additional commensalistic symbiosis of this coral‐worm holobiont is found between the *Aspidosiphon* worm and the cryptoendolithic bivalve *Jousseaumiella* sp., which resides within the cavity of the coral skeleton. To understand the morphological alterations caused by these symbioses, interspecific relationships, with respect to the carbonate structures between these three organisms, are documented using high‐resolution imaging techniques (scanning electron microscopy and µCT scanning). Documenting multi‐layered symbioses can shed light on how morphological plasticity interacts with environmental conditions to contribute to species persistence.

## Graphical abstract

The photosymbiotic solitary corals *Heteropsammia cochlea* and *Heterocyathus aequicostatus* live in symbiosis with the sipunculid worm *Aspidosiphon muelleri muelleri*, and the cryptoendolithic bivalve *Jousseaumiella* sp., which reside within the cavity of the coral skeleton. The symbiosis enables the coral holobiont to escape sediment cover in turbid conditions. High‐resolution imaging reveals morphological alterations caused by these symbioses, and allows to better understand the interspecific relationships.

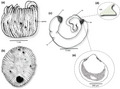

## 1 INTRODUCTION

Elevated turbidity, as well as naturally turbid conditions associated with coral reef degradation have recently gained attention, first, as potential analogues for anthropogenic sediment runoff, and second, as potential refugia from thermal bleaching events by mitigating high water temperatures in coral reefs (Sully & van Woesik, 2020). While some coral species migrate to shallower waters to overcome the effects of turbidity (Muir & Wallace, 2016), in the past years, several studies have described sediment‐resistant coral reef assemblages. For example, the coral communities of Iraq seem to shift toward slow‐growing massive coral species (Pohl et al., 2014). In the Brazilian Abrolhos Bank, a greater abundance of species was found to favor higher nutrient levels and featured sediment‐shifting capabilities (Coni et al., 2017), while in the Great Barrier Reef (GBR) of Australia, corals have been observed to shift their metabolism toward more heterotrophic lifestyles (Anthony, 2000), assumedly compensating with feeding for the lowered carbon fixation by photosynthesis (Atkinson, 2011). These turbidity‐resistant corals are characterized by massive growth forms and large polyps, and they show mechanisms for sediment removal (mucus production and transport via ciliary currents, tissue pulsation) (Bongaerts et al., 2012; Lasker, 1980; Logan, 1988; Stafford‐Smith, 1993). Also, they are adapted to the higher nutrient loads by exhibiting higher heterotrophic rates (Anthony, 2000, 2006), or by hosting lineages of *Symbiodinium* adapted to low light levels (Garren et al., 2006; LaJeunesse et al., 2010). In addition, corals exhibit unique ectosymbionts (e.g., trapeziid crabs), which aid in the removal of sediments (Stewart et al., 2006), as well as increase flow rates within interstices of coral skeletons (e.g., Doo et al., 2018).

To date, most studies on the effects of turbidity in reefs have focused on shallow‐water reef‐building colonial species (e.g., Brazil: Francini‐Filho et al., 2013; Leão et al., 2003; Loiola et al., 2013). Previous studies of solitary corals from inshore turbid settings describe the genus *Scolymia* in the family Faviidae (Coni et al., 2017), a genus that, in contrast to the free‐living species, comprises attached corals. Members of this genus are known to actively remove sediment off their surface by means of combined ciliary action and mucus entanglement (Logan, 1988; Tomascik & Logan, 1990). They are less susceptible to bleaching and subsequent mortality, and appear to prefer coastal areas with high sediment and nutrient loads, where they demonstrate high levels of heterotrophy (Coni et al., 2017). Another group in the family Fungiidae, in contrast, are free‐living and show highly developed mobility, ecomorphological adaptations such as abrasion‐resistant skeletons, passive and active hydromechanical adaptations such as burial avoidance, and the ability to right themselves with the aid of polyps (Hoeksema & Bongaerts, 2016; Hoeksema & Moka, 1989). Furthermore, time‐lapse photography of *Lobastis scutaria* and *Herpolitha limax* demonstrates their ability to remove sediment by pulsed, polyp inflation, in addition to ciliary action and mucus entanglement (Bongaerts et al., 2012). These characteristics allow for their distribution across reef‐wide environments and possibly into uncolonized areas, where they could potentially seed new reefs (Sheppard, 1981), which would be an adaptive advantage under increasing sediment loads.

In this study, we add to the growing literature documenting the symbiotic associations of two solitary stony coral species from two separate families and suborders, namely, the Dendrophylliidae *Heteropsammia cochlea* (Spengler, 1781) and the Caryophylliidae *Heterocyathus aequicostatus* (Milne Edwards & Haime, 1848). They are associated with the boring cryptic sipunculan worm *Aspidosiphon muelleri muelleri* (Diesing, 1851) and the micro‐bivalve *Jousseaumiella sp*., which resides within the sipunculan worm´s cavities. These associations were found in the neritic inter‐reef channel habitats of western Zanzibar (Tanzania). They are facultatively photosymbiotic with *Symbiodiniaceae* dinoflagellates (Hoeksema & Matthews, 2015), which thrive in euphotic conditions. These two coral species are known to host photosymbionts not only on their upper side but also on their underside, while light is transferred through their skeleton to the *Symbiodiniaceae* on their underside, thus optimizing photosymbiosis (Fine et al., 2013).

*Heterocyathus aequicostatus* was first mentioned in 1948 (Milne Edwards & Haime, 1848), and *Heteropsammia cochlea* in 1926 (van der Horst, 1926), from Tanzanian waters. The symbiotic relationship between *H*. *aequicostatus* and *H*. *cochlea* with the sipunculan worm species *Aspidosiphon muelleri muelleri*, was first described by Bouvier (1894), who interpreted this to be a commensalistic relationship. Their symbiotic relationship with the montacutid bivalve genus *Jousseaumiella sp*. was initially described under the name of *Jousseaumia* by Bouvier (1894) from Yemen, and by Bourne (1905) from Sri Lanka. Both authors interpreted it to be commensalistic, with numerous small specimens of the bivalve embedded in the skin of the posterior part of the body of the sipunculan worm, and toward the innermost coils of the worm chamber. The coral–sipunculan association has also been described by Fisk (1981, 1983) and Goreau and Yonge (1968) who reported occurrences at Wistari Reef (southern Great Barrier Reef) and Lizard Island (Australia), and by Hoeksema and Best (1991) from Indonesia. In the Western Indian Ocean, Feustal (1965) and Yonge (1975) found that the coral larva settles on shells of dead gastropods, which were already bored by sipunculan larvae, which previously settled on the shells. Similarly, Pichon (1974) described observations from Madagascar that implied that this co‐habiting type of symbiosis was initiated by the coral planula larva settling on the micro‐gastropod shell, which was already inhabited by the sipunculan. When the worm grows too large for the sheltering gastropod shell, the coral is forced to provide protection by growing around the worm. This symbiotic relationship has been described as a mutualistic one, with the worm being physically protected against predators, and the coral being transported away from being buried in sediment (George, 2012), or being stabilized in lose sediment (Fine et al., 2013). In this study, we focus on the morphological characteristics and adaptive advantages of this symbiotic relationship, which previously has not yet been described in depth.

## 2 METHODS

### 2.1 Field sampling and abundance estimation

**FIGURE 1:**
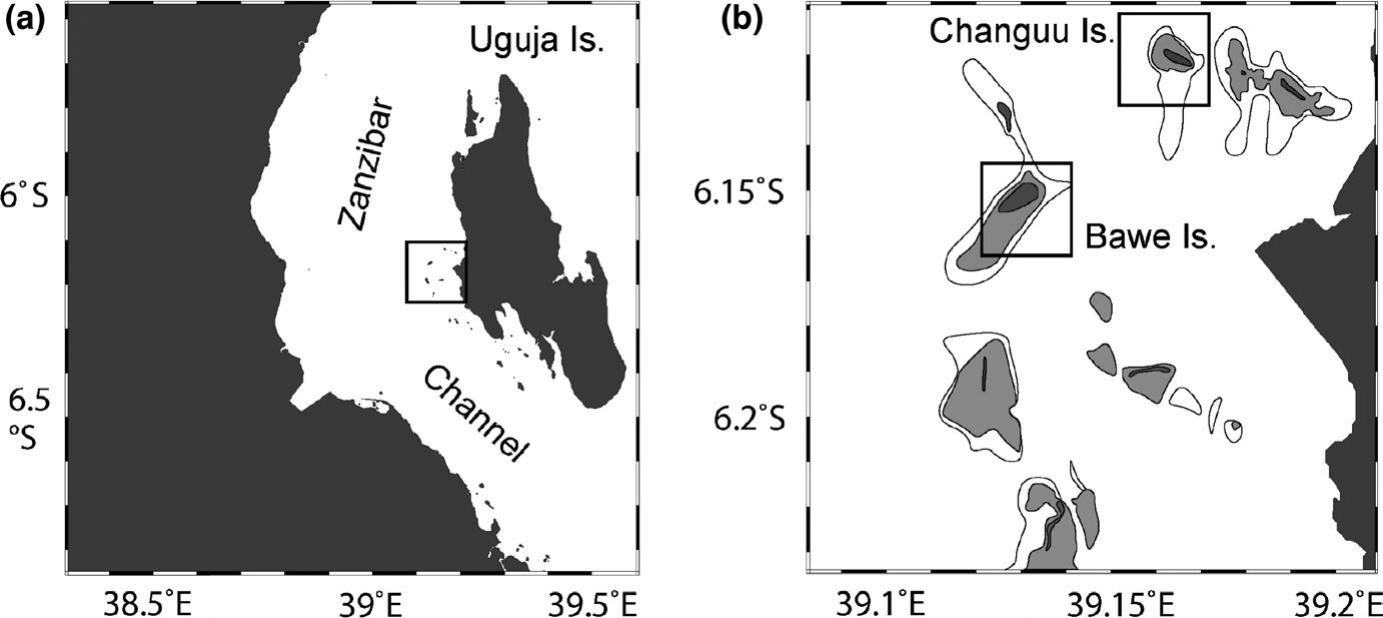
Map showing (a) the location of the study area in the Zanzibar Archipelago; and (b) the study sites (black squares indicate study sites)

The study was conducted in the vicinity of Bawe Island (6°9′19.08″S, 39°8′10.25″E) and Changuu Island (6°7′7.57″S, 39°9′58.90″E), two small reef‐fringed islands to the west of Unguja (Zanzibar) Island, located approximately 7 and 5 km, respectively, offshore of the capital, Stone Town (Figure 1). The sedimentological description, community composition, and coral cover census of the study sites can be found in Herrán et al. (2017).

Sampling took place from August to November 2014 during the slightly cooler, drier (SE monsoon) season when slightly stronger winds and a northward‐flowing surface current dominated, and the reefal waters were relatively clear. The SE monsoon is followed by an onset of the short rains, followed by the warmer and more humid NE monsoon starting in December, characterized by southward‐flowing surface currents, long rains, and slightly increased turbidity of the reefal waters.

Two areas of 150 m × 50 m were investigated by two SCUBA divers in water depth between 10 and 25 m, in order to locate the solitary corals (search and recovery method; PADI, 2003). The 20 m × 2 m belt transect method (English et al., 1997) was used, with three replicates at each site, to determine the population density (individuals/m^2^) of *H*. *cochlea* and *H*. *aequicostatus*. At Changuu, sampling took place in the tidal channel south of Changuu Island (27 counts of *H*. *cochlea*, two specimens recovered, sample IDs: Hc1a, Hc1c). At Bawe, the sampling took place in the forereef (97 counts; 61 *H*. *cochlea* and 36 *H*. *aequicostatus*; four specimens recovered, sample IDs: *Hc1b,* Ha1a, Ha1b, Ha1c). Sample details are given in Table 1. In addition, skeletal remains of *H*. *aequicostatus* (two adults, four juveniles, i.e., nubbins) and *H*. *cochlea* (one adult, 11 juveniles) were collected by SCUBA divers from the seafloor sediment in Changuu for thin section analysis.

**TABLE 1:** Coral specimens (*N* = 6) collected in the vicinity of Bawe and Changuu reefs

Sample ID	Species	CT scan and 3D model	Calice Length long axis (mm) SD ± 0.1	Calice Length short axis (mm) SD ± 0.1	Height (mm) SD ± 0.1	Weight (g) SD ± 0.001	Number of polyps	Num‐ber of pores	Sampling location, substrate
Hc1a	*H. cochlea*		17.7	10.1	17.4	5.24	1	8	Changuuvery fine sand
Hc1b	*H. cochlea*		Calice 1: 10.4 Calice 2: 8.7	Calice 1: 10.3 Calice 2: 8.6	12.5	10.16	2	3	Bawe fine pebbles
Hc1c	*H. cochlea*	x	13.3	7.3	15.0	5.08	1	10	Changuuvery fine sand
Ha1a	*H. aequi‐costatus*		12.5	10.8	10.4	2.87	1	7	Bawe fine pebbles
Ha1b	*H. aequicostatus*		11.5	10.3	8.9	3.25	1	8	Bawe fine pebbles
Ha1c	*H. aequi‐costatus*	x	11.7	9.6	9.7	2.88	1	9	Bawe fine pebbles

### 2.2 Culturing in the aquarium facility

The six live‐collected corals were stored in seawater aquaria at the Institute of Marine Sciences, Zanzibar, in order to maintain the specimens in the physical conditions closest to that at the sites of collection, before transporting them to ZMT in Bremen, Germany. Upon arrival at ZMT, the corals were placed in a seawater aquarium for three weeks for photographic documentation.

### 2.3 Micro‐structural analyses of the solitary corals

For structural analyses and µCT scans of the live‐collected corals, two coral specimens (one *H*. *cochlea* and one *H*. *aequicostatus*, see Table 1) were fixed in 40 ml of 99.8% Ethanol. Afterward they were treated with 90% H_2_O_2_ for 48 hours to remove organic matter and dried for 12 h at 40°C. A stand‐alone micro‐CT (µCT) scanner (Skyscan 1172) was used at 180 KeV to study the 3D structure of the whole carbonate skeleton. The µCT scans provide a nominal resolution of 5–8 µm per voxel, depending on magnification scale, and were scanned at angular increments of 0.9° rotation steps over a period of 3–11 h.

The coral skeletons were then embedded in epoxy and sliced through the center along their long edge for SEM analysis. The sections were polished to approximately 35 µm thickness and gold sputtered. SEM images of the thin sections were taken with a Tescan^®^ Vega 3 XMU SEM at 20 kV (SE detector). Backscattered electron (BSE) and secondary electron (SE) images were taken at 15 kV in low‐vacuum mode.

Light microscopy was undertaken on thin sections cut from the skeletal remains collected from the sediment with a Keyence VHX‐ 5000 equipped with a VH‐Z20R lens a VHX‐ 5020 camera and XY‐Stage VHX‐S550E.

## 3 RESULTS

### 3.1 Field observations

Solitary corals were identified *in* *situ* from water depths between 16 and 21 m below sea‐level from the windward‐fringing reef flanks, which extends 1.5 km off the southern side of Bawe Island. *Heteropsammia cochlea* was dominant (0.51 individuals/m^2^), followed by *Heterocycathus aequicostatus* (0.30 individuals/m^2^), with an associated error of <10%. At Changuu Island, solitary corals were scarce, and only a few specimens of *H*. *cochlea* restricted to the eastern tidal channel (about 0.22 individuals/m^2^) were identified, while no *H*. *aequicostatus* were observed here.

The development of two polyps by so‐called budding was observed at this location (Changuu Island) in the 38%, *N* = 27 of the specimens of *H*. *cochlea*. A total of 3%, *N* = 27 of *H*. *cochlea* showed even more than two polyps. Budding is a feature common in this species and is usually developed by elongation and later bipartition of the main corallite (Arrigoni et al., 2014).

### 3.2 Live‐collected specimens

**FIGURE 2:**
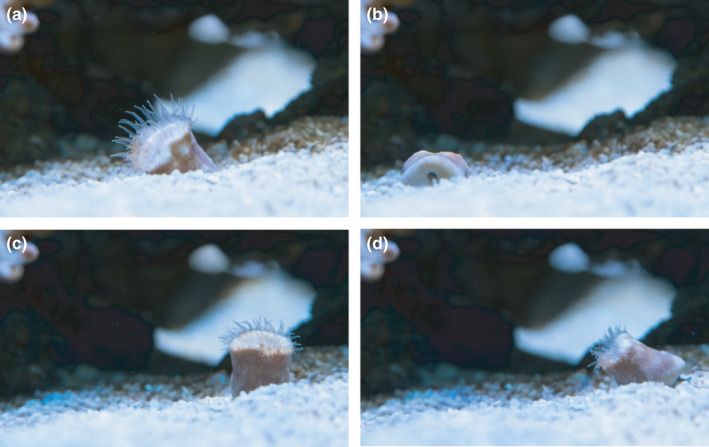
Screen shots of video of “walking” *Heteropsammia cochlea* cultured in the aquarium facility at the Leibniz Centre for Tropical Marine Research (ZMT), Bremen, Germany. Between (a) and (b), 8 min have passed, (c) was taken some 20 min later, and finally (d) another 3 min later. Note that in (b) the bottom of the coral with the opening for the sipunculan is visible. For full time lapse video see Video S1

The live‐collected specimens thrived in the aquarium culture at ZMT, and the moving (“walking”) behavior was observed (see video in Figure 2).

**FIGURE 3:**
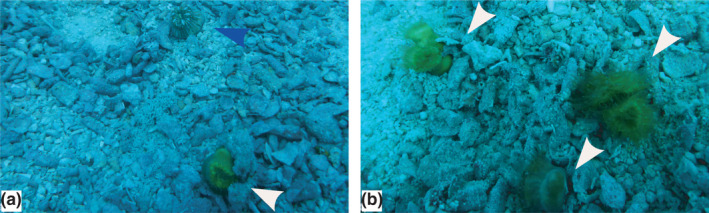
Solitary corals at Bawe Island, Zanzibar, showing: (a) *Heterocyathus aequicostatus* (blue arrow) and *Heteropsammia cochlea* (white arrow). (b) *Heteropsammia cochlea* (white arrows), the upper two with bipartite corallites. Field of view: app. 0.3 × 0.15 cm; depth = 16–19 mbsl

**FIGURE 4:**
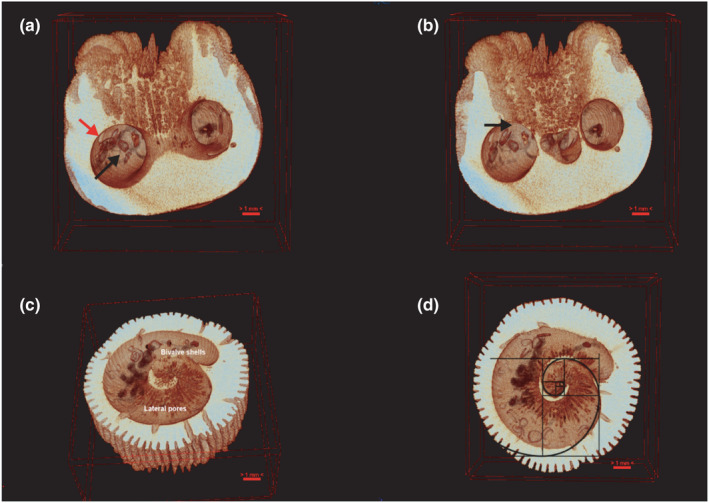
The µCT scans of *Heterocyathus aequicostatus* in an upright position: (a) vertical section, red arrows to indicate the cavity in which the sipunculid worm resides, and black arrow that point to multiple *Jousseaumiella* sp. specimens; (b) the interference of the *Aspidosiphon* chamber on the *Heterocyathus* septum and columella (black arrow); (c) oblique and (d) horizontal sections of the *Aspidosiphon* chamber, revealing the helical shape of the cavity

The calice length of the three specimens collected of *H*. *aequicostatus* ranged in their long axis from 11.7 mm to 12.5 mm (Table 1). The color of their organic tissues ranged from pale brown to dark brown (Figure 3). The coral skeletons were sub‐circular with a slightly convex base (Figure 4). Edges of the corallum were smooth, with a roundish and approximately 0.5 mm deep calice. The imperforate theca showed one axis growing septa along the vertical axis, which was ornamented with a spike‐like and granulate texture.

The three specimens of *H*. *cochlea* showed a squat base, and a flat and oval calice where the polyp emerged. The narrowed and sigmoidal calice ranged in its long axis length from 8.7 mm to 17.7 mm. One of the specimens of *H*. *cochlea* showed two corallites, that is, budding.

All coral specimens showed an aperture of ca. 1 mm in diameter, and several lateral pores—or foramina—along the aboral side (Figures 4 and 6), which indicates that they lived in symbiosis with a sipunculan worm of the genus *Aspidosiphon*.

**FIGURE 5:**
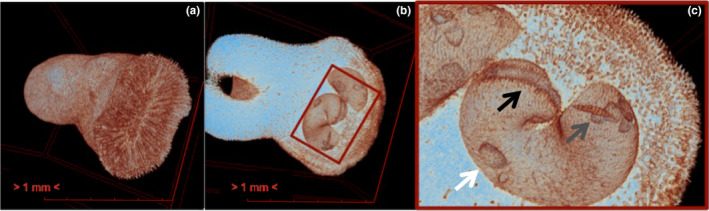
The µCT scans of *Heteropsammia cochlea* showing the: (a) frontal view of the specimen; (b) ventral (intermediate) layer, revealing bivalve shells on the walls; (c) detail of the *Jousseaumiella* specimens (white arrow), dwelling in the *Aspidosiphon* chamber, with an unknown artifact (grey arrow), and evidence of scratched walls (black arrow)

The µCT analyses of both species showed the morphological expression of the three organisms involved, the coral, the sipunculan, and the bivalve (Figures 3 and 4). The ellipsoidal cavity created by *Aspidosiphon*, the so‐called chamber, sweeps through the coral frame in a spiral pattern (Figure 4), in which proportions met similarities to the aurea spiral in the innermost parts. We observed exclusively in *Heterocyathus aequicostatus* that the *Aspidosiphon* chamber was in contact with the living tissue (mesenteries) of the coral polyp, allocated in the columella (Figure 4). The µCT of *H*. *cochlea* reveals internal crevices and internal scratch‐like structures (Figure 5) on the walls of the original chamber, which we interpret to be the imprint of the introvert of the sipunculan on the walls of the *Aspidosiphon* chamber. There appears to be a change in morphology in the coral skeleton in response to the sipunculan worm implying that the worm could be taking waste resources from the coral. In the µCT, no original substrate of the coral larvae was identified in either species.

**FIGURE 6:**
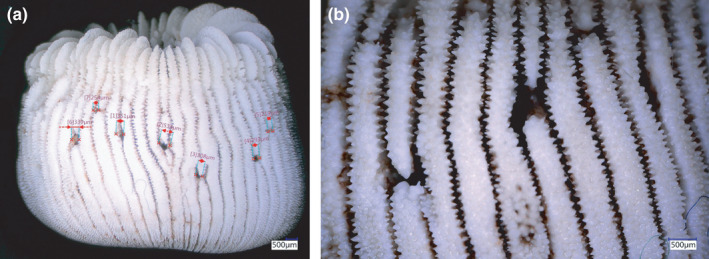
Outside view of the skeleton of *Heterocyathus aequicostatus*: (a) overview featuring numerous aligned foramina connecting the chamber of *Aspidosiphon* with the outside environment; (b) close up of foramina

The lateral pores observed in the coral skeletons form a linear pattern along the middle axis of the *Aspidosiphon* chamber (Figure 6). The diameter of the pore openings in the *H*. *aequicostatus* specimens average at 328 ± 94 μm (number of pores measured is *N* = 24). The peritheca and costae growth follows the vertical axis. In the *H*. *cochlea* specimens, the foramina were smaller and less abundant (*N* = 21, average diameter 285 ± 86 μm).

**FIGURE 7:**
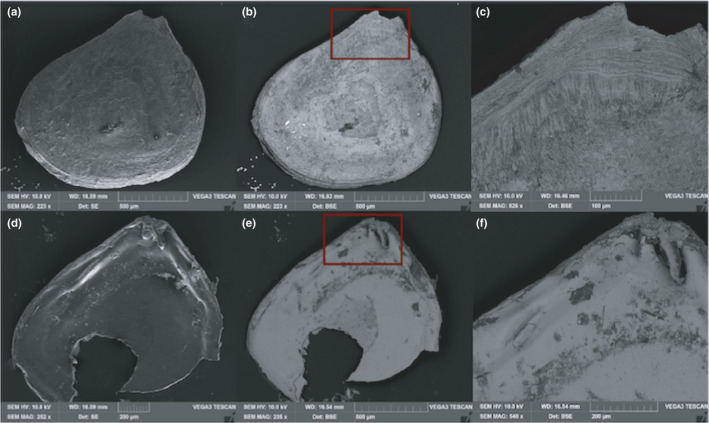
SEM images of *Jousseaumiella* spp. showing the: (a) secondary electron (SE) images of exterior left valve: length = 1328 µm, height = 1191 µm; (b) backscatter electron image (BSE) from the exterior left valve; (c) close up of the beak; (d) SE image of the interior right valve; (e) BSE image of the interior valve; and the (f) close up of the dorsal side of the valve showing the hinge

In the cavity of *Aspidosiphon* in both of the corals studied by µCT, numerous bivalves of 0.8 to 1.0 mm in length were observed. In the cavity of *H*. *aequicostatus* (Figure 4), 14 individuals of the bivalve were recognized, while 10 bivalves were identified in the cavity of *H*. *cochlea* (Figure 5). As seen under SEM, the bivalves were bilaterally symmetrical, with a heterodont hinge lying in the sagittal plane. They featured two forms of hinge teeth of different sizes, with a shorter anterior ill‐defined tooth and a posterior cardinal tooth, closely curving posteriorly (Figure 7). This bivalve is identified as *Jousseaumiella* spp., which has previously been observed to live exclusively in commensal associations with sipunculans residing within *H*. *aequicostatus* and *H*. *cochlea* (Bourne, 1906). Bourne (1906) identified two species, namely, *J*. *heterocyathi*, which was only found in *Heterocyathus*, and *J*. *heteropsammiae*, which was only found in *Heteropsammia*. Herein, we did not identify the bivalve down to the species level.

### 3.3 Sedimentary skeletal remains

**FIGURE 8:**
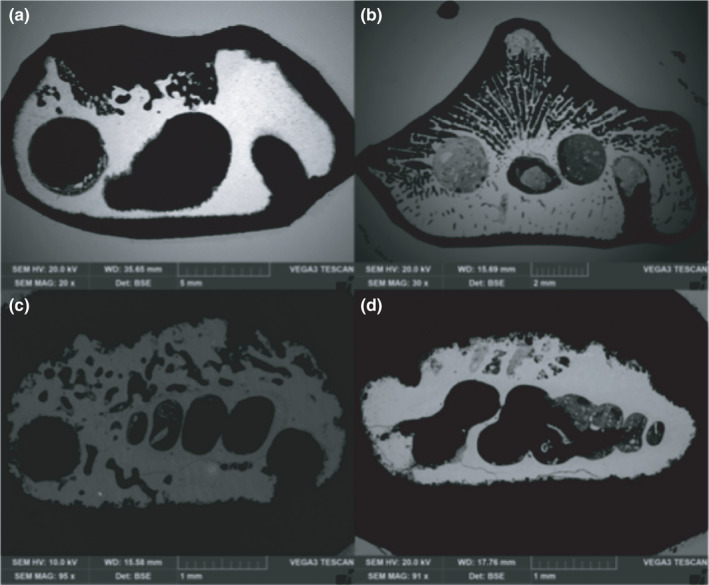
Thin section images of the sedimentary material with: (a) the remains of an adult *Heterocyathus aequicostatus*, lacking signs of an initial substrate; (b) a skeleton of a juvenile *Heteropsammia cochlea*, with remains of the gastropod shell used as the initial substrate, and showing the internal cementation; and (c), (d) the remains of gastropod shells located in the middle axis of the skeleton of nubbin stages of *H*. *aequicostatus* and *H*. *cochlea*, respectively

Thin section images of the adult specimens from the sedimentary material are devoid of the original substrate (Figure 8a). In contrast, the remains of juveniles reveal gastropod shells as initial substrate and internal cementation on this substrate. The gastropods are located in the middle axis of the nubbin skeletons (Figure 8b–d).

## 4 DISCUSSION

The majority of studies have previously described the symbiosis between the coral and sipunculan worm as being mutualistic, rather than parasitic (Fisk, 1981, 1983; Goreau & Yonge, 1968; Hoeksema & Best, 1991; Igawa et al., 2017). Our findings, further support this interpretation on the basis of the fact that the coral tissue was not affected by the lateral pores created by the sipunculan. In addition, the effect of etching of the *Aspidosiphon* chamber within the coral was minor (Figure 5), implying that the coral grows around the worm, rather than the worm boring into the coral skeleton (cf. Igawa et al., 2017). This interpretation is further supported by the observation, from the sedimentary remains, that the coral grows around a gastropod shell, which already features an *Aspidosiphon* chamber (Figure 8). Previous studies have found traces of the original substrate, such as a gastropod or scaphopoda shell within live specimens (Fisk, 1981, 1983; Gill & Coates, 1977; Goreau & Yonge, 1968; Stolarski et al., 2001; Zibrowius, 1998). Since the nubbin stages from the sediment reveal gastropods as substrate (Figure 8), we speculate that dissolution, reabsorption, or remineralization of the primary carbonate structure of the gastropod shell has taken place.

Skeletal properties of corals are documented to exhibit high plasticity through modification of their morphology, growth, and array of symbiotic associations to adapt to a wide range of ecological conditions and to maximize fitness. The extensively studied relationship between corals and their unicellular dinoflagellate endosymbionts (*Symbiodiniaceae*), results in high productivity benefits such as enhanced growth and calcification rates, increased metabolism, and respiration. In turn, endosymbionts receive a substrate and protection. In mutalistic (++) relationships, both partners maintain benefits that exceed the cost (Axelrod & Hamilton, 1981). However, symbiotic interactions between mushroom corals and various other invertebrate and fish species, can be commensal (+0) or parasitic (−+), both of which may have been evolutionary driving forces behind current symbiotic mutualistic partnerships. For example, cryptobenthic species associated with mushroom corals include fish (e.g., *Eviota*) and shrimp (e.g., *Cuapetes*) living on, and acoelomorph flatworms (e.g., *Waminoa*) living in the coral; gall crabs (e.g., *Fungicola*), hermit crabs (*Diogenes*), serpulid tube worms, and brittle stars (e.g., *Ophiotrix*), endolithic/‐parasitic boring gastropods (e.g., *Leptochonchus*), and mussels (*Lithophaga*) (Bos & Hoeksema, 2017; Gittenberger & Gittenberger, 2011, and references cited therein; Hoeksema et al., 2018; Igawa et al., 2017). The nature of many of these relationships is not clear, but associated fauna may benefit from receiving shelter or food particles from their host (Bos & Hoeksema, 2017; Stewart et al., 2006), as well as increasing resilience toward increased temperature and CO_2_ levels (Doo et al., 2018).

**FIGURE 9:**
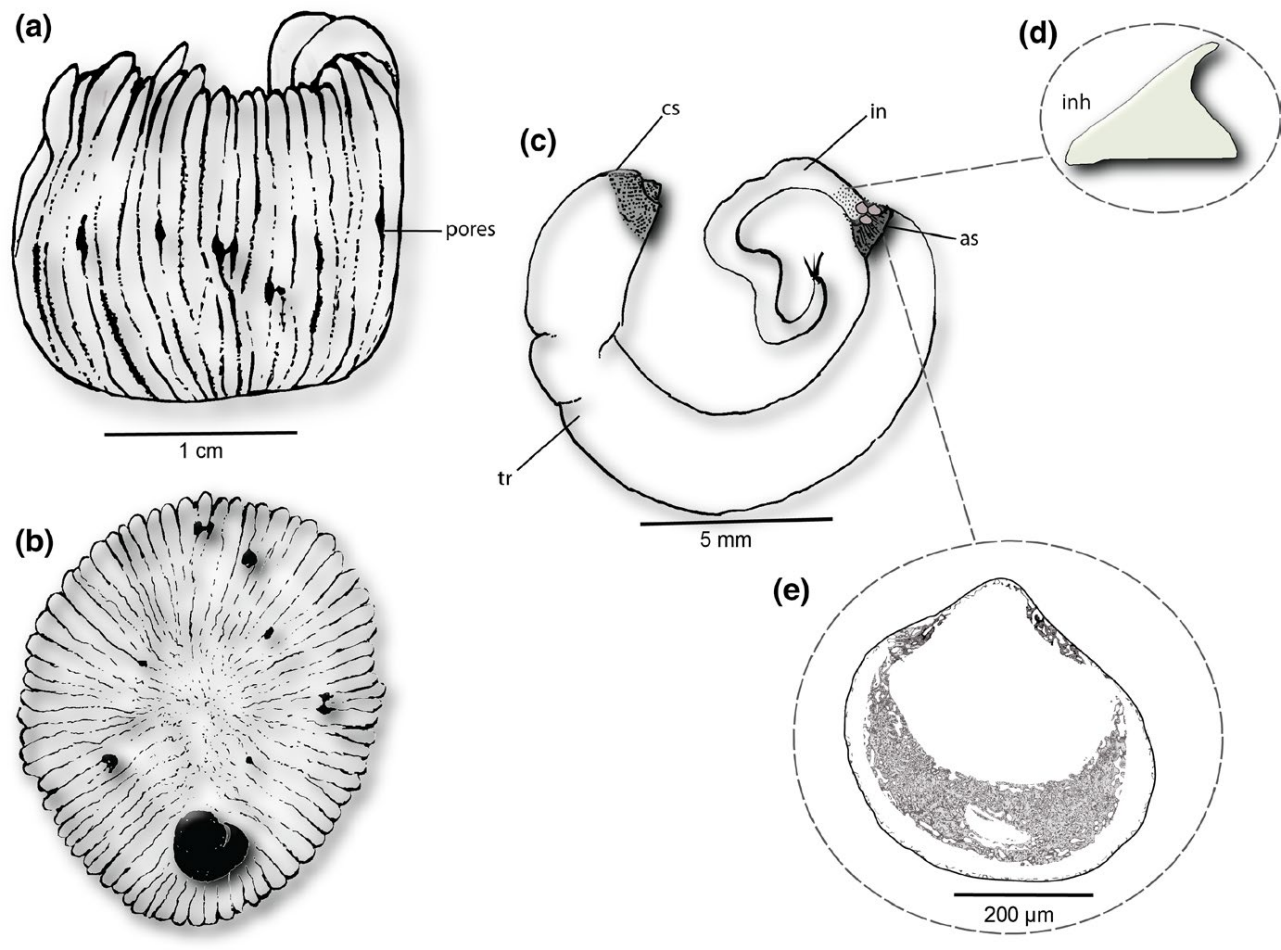
A schematic sketch of the three‐way symbiotic relationship between: a generalized solitary coral (e.g., *Heterocyathus*), showing (a) lateral and (b) aboral views; (c) a sipunculan worm (e.g., *Aspidosiphon*), showing (d) a unidentate hook structure; (e) and an endolithic bivalve (e.g., *Jousseaumiella*)

The complex and apparently mutualistic symbiosis between the dinoflagellate endosymbiont solitary coral species (*H*. *cochlea* and *H*. *aequicostatus*), the sipunculan worm (*A*. *muelleri*), and the bivalve (*Jousseaumiella* spp.) (Figure 9), raises the question of what, if any, is the benefit to these different organisms involved. If this is a successful relationship, then why is it so rare, compared to the typical symbiotic producer‐in‐consumer relationship between dinoflagellate endosymbionts and reef‐building coral taxa? One hypothesis is that this consumer‐in‐consumer‐type symbiosis is less frequently encountered, because it occurs across a wide range of different environments, across varying nutrient and photic conditions, and possibly within areas of greater heterotrophic plasticity.

This unique coral–sipunculan worm association exemplifies convergent coevolution (Hoeksema & Best, 1991; Igawa et al., 2017). *Heterocyathus aequicostatus* and *Heteropsammia cochlea* belong to two different families (Caryophylliidae and Dendrophylliidae, respectively), implying that this symbiosis developed independently as an example of convergence (Hoeksema & Best, 1991). A recent phylogenetic study suggests that the sipunculan worms comprise two distinct clades, both of which are associated with both of the coral species, thus this association is not species‐specific (Igawa et al., 2017). In support of coevolution, the worm morphology shows plasticity, determined by the internal structure of the coral host. As the worm lives and grows within the coiled chambers of the coral, the coral chambers grow simultaneously around it, resulting in “lodging mutualism“ (Igawa et al., 2017). In another associative case, for example, the sipunculan worm also inhabits polychaete worm tubes; however, as the sipunculan worm grows and fills the tube, it no longer fits and needs to move into a larger worm tube.

While the interaction between *Heteropsammia* sp. and *Aspidosiphon* has largely been interpreted as commensalistic, Arnaud and Thomassin (1976) mention that they found the date mussel *Lithophaga lessepsiana* to bore into *H*. *cochlea* (then: *H*. *michelini*) just above the *Aspidosiphon* chamber in a complex relationship that they interpret as parasitic as it harms the coral host. Even though there are recent reports that suggest a harmful association with serpulid worms for the host (Hoeksema et al., 2019), the majority of symbioses between corals and worms seems to be advantageous across a range of environments, and particularly in deep‐sea conditions. For example, the colonial scleractinian cold water coral *Desmophyllum pertusum* has been observed to live in association with the polychaete worm *Eunice norvegica* in the deep waters of the North East Atlantic (Mueller et al., 2013). This association was interpreted to be a mutualistic symbiosis, where the polychaete positively stimulates calcification of the coral by up to four times. In turn, the coral provides substrate and shelter but also increases fitness by improving tissue assimilation and food partitioning for the worm (Mueller et al., 2013). In addition, aquaria observations suggest that *E*. *norvegica* benefits by stealing food from the coral host, while at the same time, it cleans the coral's framework of detritus and protects it from predators using aggressive behavioral displays (Buhl‐Mortensen & Mortensen, 2004; Mueller et al., 2013).

Associations of a similar kind have been found in the geological past. Throughout the Cenozoic, fossil corals are reported to have lived in symbiosis with a sipunculan worm (Stolarski et al., 2001). In the Cretaceous, *Heterocyathus priscus* is thought to have lived in symbiosis with a possibly sipunculan worm, while the Devonian tabulate coral *Pleurodictyum problematicum* is interpreted to have provided protection to its associated worm *Hicetes* sp. (Darrell & Taylor, 1993; Gerth, 1952; Stolarski et al., 2001). The significance of these associations lies in better understanding how mutual facilitation can enhance ecosystem functioning and species persistence under changing environmental conditions.

The greatest advantage and functionality of this coral‐worm association may be particularly important in deeper mesophotic, turbid, sediment‐laden, soft‐bottom environments, which are rather unfavorable for most photosymbiotic benthic reef‐dwellers. However, these associations typically occur in shallow, relatively clear water, coarse‐grained, and inter‐reef environments. The worm feeds on organic matter, the removal of which is well known to promote the recycling of nutrients. We speculate that the removal of detritus by the worm from the coral tissue and from the engulfing water layer, could also play an important role, similar to those observed in other ecosymbiotic organisms (e.g., porcelain crabs). Another ecosystem function of the worm could be to stabilize the sediment by removing fine‐sized organic‐rich sediment particles.

This corroborates the findings presented in previous literature where a connection between the coral–sipunculan symbiosis is more prevalent in turbid conditions. The coral and sipunculan partners benefit particularly from this symbiosis by the fact that the coral can be moved over the sediment surface by the feeding worm (Bouvier, 1894; Feustel, 1965; Fisk, 1981, 1983; Gill & Coates, 1977; Goreau & Yonge, 1968; Hoeksema & Best, 1991; Yonge, 1975). The corals’ internal morphology supports a hollow skeleton, which can be dragged around by a sipunculan worm above the soft bottom, and a squat flat base that is stable enough to be anchored under high energy conditions. Fine et al. (2013) observed that the sipunculan prevents burying of the coral and can anchor the coral in the substrate under strong tidal current conditions. This seems to conform well with the observations that were made in Zanzibar. It has also been shown that *Heteropsammia* and *Heterocyathus* rely on preying on zooplankton, and the sipunculan worms move them out of sediment enabling them to feed (Mehrotra et al., 2016).

Fine et al. (2013) also found that this symbiosis also occurred in the nonturbid waters of the Great Barrier Reef, Australia, where the holobionts appeared to occupy unstable coarse sand bottoms. Fine et al. (2013) hypothesized that, in the coral‐derived carbonate sands, optical conditions were ideal for the dinoflagellate endosymbionts of the corals to optimize light‐trapping for photosynthesis on the buried underside of the coral. Thus, apparently this symbiosis potentially allows for adaptation to one of the two, or both, turbid and unstable sandy conditions.

The coral–worm mutual symbiosis may increase the corals’ resilience, particularly under changing and episodically turbid conditions, as found in the reefs and inter‐reef habitats off western Zanzibar. Here, they mainly occur in deeper waters, but not exceeding 20 m. In these reef channels, particles were commonly in suspension in the water column, with limited settling, while substrates were characterized as relatively clean, composed of unconsolidated, medium to very coarse, poorly‐sorted biogenic carbonates grains, and very little fines. At our study site, anthropogenic disturbances included overfishing (Lokrantz et al., 2009), port, and channel dredging activities and land‐based (untreated sewage) pollution (Moynihan et al., 2012), most of these were related to rapid population growth and tourism (Lange & Jiddawi, 2009). Sediment fluxes were known to range from 0.2 to 41.5 mg cm^2^ d^−1^ (Muzuka et al., 2010), and thus peak above threshold limits of many scleractinian corals, which is >10 mg cm^−2^ d^−1^ (Rogers, 1990). Obviously *H*. *aequicostathus* and *H*. *cochlea* are morphologically adapted to withstand variable sediment fluxes, episodic low water quality (nutrient influx), and moderate to strong currents.

Stolarski et al. (2001) and Zibrowius (1998) subdivided the coral–sipunculan association in two morphological subclasses: (1) polyporous with numerous openings or pores at the lower sides, or in the flattened base of the coral; and (2) monoporous with a single pore opening, plus a main opening. In this study, we only encountered polyporous specimens. Nonetheless, the pores were not found at the base of *Heterocyathus* spp. as described by Stolarski et al. (2001), but above the sediment–water interface, where in living position the base of the coral extended into the sediment. Such a position is presumably controlled by the sipunculan that controls the openings facing the sediment–water interface where gradients in physical–chemical properties are suitable (Santschi et al., 1990). The functionality of these pores has been extensively reviewed (Fisk, 1981, 1983; Gill & Coates, 1977; Goreau & Yonge, 1968; Stolarski et al., 2001; Zibrowius, 1998). Two main functions have been discussed, namely, the circulation of water (Feustel, 1965; Ikeda, 1922), and the release of excrements of the sipunculan (Semper, 1880; Sluiter, 1902). Other potential functions have been discussed to include the housing of unknown boring organisms (Schindewolf, 1959), or the release of nematocysts as protection measures by the sipunculan (Bourne, 1906). Stolarski et al. (2001) proposed that the origin of the polyporous morphology could be a perforation by the sipunculan using “minute asperities which beset the proboscis” (Tenison−Woods, 1880: p. 298); or that the pores were formed by the coral, when growing around extensions of the sipunculan (Jousseaume in Bouvier, 1894). Cutler (1965), however, pointed out that these appendices seemed not to exist. A third hypothesis states that the pores were pinched off from the orifice during growth (Sluiter, 1902), while a fourth one proposes that they were bored into the corallum by some other organism (Schindewolf, 1959). Chemical dissolution was excluded by Jousseaume in Bouvier (1894) and by Schindewolf (1959). Most likely, however, it seems that the coral actively overgrows the substrate, except for an efferent pore for the expandable introvert of the worm. With time, the worm continues to grow in a spiral pattern around the coral's base, and with continued growth, the coral coenosteum maintains a full cover for the worm by adaptive extra calcification (see also Beuck et al., 2007). Polyporus corolla, thus, document a periodical re‐orientation of the efferent pore in a dynamic growth interaction between the host and the symbiont organism (Cairns, 2001). The ontogenetically older pores are in many cases arranged in a roughly linear pattern, and usually show reduced diameters as compared to the polyporus corolla. They are thought to functionally assist in the facilitation of fluid exchange and in respiration (Moseley, 1881).

While in the symbiosis described herein, the coral seems to grow around the sipunculan, *A*. *muelleri muelleri* generally is able to excavate its protective home in calcareous substrate by boring into dead coral skeleton, carbonate rocks, including submerged archaeological objects (Antonelli et al., 2015; Rice, 1969). This excavating behavior differs largely from mutualistic interaction between the live coral–sipunculan consortium as discussed above. The anatomy of *A*. *muelleri muelleri* follows the characteristic sipunculan body plan (Figure 9) comprising a thickened posterior trunk and a narrower anterior introvert that can be retracted into the trunk (Rice, 1993). It is the expandable introvert that enables the mobility of the entire consortium. The introvert itself bears the tentacle crown and the mouth. A common feature in the Aspidosiphonidae is the possession of cuticular elaborations or projections such as hooks, spines, and papillae on the introvert. It is assumed that the hooks and spines mechanically support the bioerosion process (Rice, 1969, 1993) by scraping‐off biochemically etched crystalline bonds through chelating agents or acids (Williams & Margolis, 1974).

The third player in the coral–sipunculan consortium is the small galeommatid bivalve, assigned as *Jousseaumiella*, of which three species are listed by WoRMS (2021); *J*. *concharum*, *J*. *heterocyathi*, and *J*. *heteropsammiae*. Species of the Galeommatidae are known as ecto‐, or endoparasites, or commensals on many invertebrate taxa (Bristow et al., 2010; Goto et al., 2011), often linked with an intimate species–species connection. We did not find *Jousseaumiella* specimens in life position due to the methodology applied. Previous observations, however, indicate a mutualistic relationship with the sipunculan worm. The tiny and flattened bivalves live in the interspace between the sipunculan trunk and the coral housing, thus having access to the nutrient‐ and food‐enriched water flow from the outside environment and from the provision of ingredients provided by the excrements of the sipunculan to the bivalves. Fixation via byssal attachment is thought to take place on the sipunculan, or potentially on the excavated wall of the coral.

High resolution imaging techniques provide an excellent tool for increasing our understanding of the morphological co‐adaptations and beneficial traits found in mutualistic, consumer‐in‐consumer, and symbiotic‐type associations. It is unknown how these symbiotic interactions will be further influenced by environmental changes and what affect this will have on the ecosystems in which they occur. Therefore, future studies should take into consideration the array of organisms associated with corals, which contribute to their productivity, functionality, and protection.

## CONFLICT OF INTEREST

The authors declare no conflict of interest.

## AUTHOR CONTRIBUTIONS

**Natalia Herrán:** Conceptualization (equal); Formal analysis (equal); Investigation (equal); Methodology (equal); Writing – original draft (lead); Writing – review & editing (supporting). **Gita R. Narayan:** Data curation (supporting); Formal analysis (supporting); Investigation (equal); Methodology (supporting); Visualization (equal); Writing – original draft (supporting); Writing – review & editing (equal). **Steve S. Doo:** Conceptualization (supporting); Formal analysis (supporting); Investigation (supporting); Writing – review & editing (equal). **André Klicpera:** Conceptualization (supporting); Formal analysis (supporting); Investigation (supporting); Methodology (supporting); Writing – original draft (supporting); Writing – review & editing (supporting). **André Freiwald:** Conceptualization (equal); Formal analysis (supporting); Investigation (supporting); Methodology (supporting); Writing – original draft (supporting); Writing – review & editing (supporting). **Hildegard Westphal:** Conceptualization (equal); Formal analysis (supporting); Funding acquisition (lead); Investigation (supporting); Methodology (supporting); Project administration (lead); Resources (lead); Writing – original draft (supporting); Writing – review & editing (lead).

## Supporting information

Video S1

Supplementary Material

## Data Availability

Raw data of transects for abundance data *H*. *cochlea* and *H*. *aequicostatus* are available in Dryad (https://doi.org/10.5061/dryad.ksn02v761).
