# Supplementary material for: High‐resolution imaging sheds new light on a multi‐tier symbiotic partnership between a “walking” solitary coral, a sipunculan, and a bivalve from East Africa
Source: Ecol Evol. 2022 Mar 8;12(3):e8633. doi: 10.1002/ece3.8633 (PMC8928893; doi:10.1002/ece3.8633)
Supplement: Supplementary file 2 — Supplementary Material [file ECE3-12-e8633-s001.doc]

„Supplementary Material 1: Time lapse video of “walking” *Heteropsammia cochlea* cultured in the aquarium facility at the Leibniz Centre for Tropical Marine Research (ZMT), Bremen, Germany. The video has been edited at 25 frames per second and with one picture taken every 10 seconds.“

https://zmtcloud.leibniz-zmt.de/index.php/s/htRMbTXhGBuLz9V
